# ﻿*Ilyocyprisleptolinea* Wang & Zhai, sp. nov., an ostracod (Ostracoda, Crustacea) from the late Quaternary of Inner Mongolia, northern China

**DOI:** 10.3897/zookeys.1137.94224

**Published:** 2022-12-22

**Authors:** Qianwei Wang, David J. Horne, Jiawei Fan, Ruilin Wen, Robin J. Smith, Min Wang, Dayou Zhai

**Affiliations:** 1 Yunnan Key Laboratory for Palaeobiology, Institute of Palaeontology, Yunnan University, Kunming 650500, China; 2 MEC International Joint Laboratory for Palaeobiology and Palaeoenvironment, Yunnan University, Kunming 650500, China; 3 School of Geography, Queen Mary University of London, Mile End Road, London E1 4NS, UK; 4 Institute of Geology, China Earthquake Administration, Beijing 100029, China; 5 Key Laboratory of Cenozoic Geology and Environment, Institute of Geology and Geophysics, Chinese Academy of Sciences, 19 Beitucheng West Road, Chaoyang District, Beijing 100029, China; 6 CAS Center for Excellence in Life and Paleoenvironment, Beijing, 100044, China; 7 Lake Biwa Museum, 1091 Oroshimo, Kusatsu, Shiga 525-0001, Japan; 8 Yunnan Key Laboratory of Plateau Geographical Processes and Environmental Changes, Faculty of Geography, Yunnan Normal University, Kunming 650500, China

**Keywords:** Fossil, lacustrine sediment, marginal ripplet, new species, outline analysis, Xiaojinggou Basin

## Abstract

*Ilyocyprisleptolinea* Wang & Zhai, **sp. nov.** is described from late Quaternary sediments in central-eastern Inner Mongolia, northern China. The new species, which has a carapace shape and pitted surface typical of the genus, is characterised by double rows of fine, densely arranged marginal ripplets, separated by an inner list, along both anterior and posterior calcified inner lamellae in the left valve. Outline analysis and Principal Component Analysis indicate that its morpho-space overlaps with *I.bradyi* Sars, 1890, *I.japonica* Okubo, 1990, and *I.mongolica* Martens, 1991, which have living or fossil representatives in Inner Mongolia, but it is clearly discriminated from *I.innermongolica* Zhai & Xiao, 2013. Judging from the relatively coarse lithology dominated by silt and sand, and the lack of accompanying brackish-water ostracods, *I.leptolinea* Wang & Zhai, **sp. nov.** may have lived in a relatively shallow freshwater lake. It perhaps can be added to the list of species that went extinct during the Quaternary, but the timing and process of extinction await further investigation.

## ﻿Introduction

The Ostracoda genus *Ilyocypris* Brady & Norman, 1889, dating back to the Late Cretaceous (Yang 1981; Hou et al. 2002), has a rich, worldwide fossil record. There are at least 40 known extant species: 38 species included in the checklist of Meisch et al. (2019), plus two more erected since by Smith et al. (2019) and Peng et al. (2021). *Ilyocypris* species thrive in various non-marine aquatic habitats, including lakes (e.g., *Ilyocyprissalebrosa* Stepanaitys, 1960, cf. Smith et al. 2011), ponds (e.g., *Ilyocypristibeta*Peng et al., 2021), swamps (e.g., *Ilyocyprisbradyi* Sars, 1890, cf. Meisch 2000), and running waters (e.g., *Ilyocyprisdentifera* Sars, 1903, cf. Smith et al. 2019). Some members of this genus are adapted to artificial environments, such as lotus fields (e.g., *Ilyocyprisjaponica* Okubo, 1990, cf. Smith et al. 2019) and rice fields (several species, see Smith et al. 2018), and the eggs and/or living individuals of some species are desiccation-resistant (e.g., *Ilyocyprisangulata* Sars, 1903 and *I.dentifera*).

Despite the above-mentioned ecological disparity and taxonomic diversity within *Ilyocypris*, the morphological differences between many of its species are so subtle that reliable species identification can be difficult without information of the male reproductive organs (Smith et al. 2019). For palaeontologists who work on valve material, the general similarity of valve morphology within the genus on one hand, and the intra-species variability of the nodes on the other hand, have further complicated the taxonomy (Meisch 2000; Smith et al. 2019).

With the help of Scanning Electron Microscopy (SEM), van Harten (1979) observed the ‘marginal ripplets’ on the postero-ventral region of the calcified inner lamella of the left valve of *Ilyocypris*, and Janz (1994) proposed that this character could be useful for species identification. Some later works (Mischke et al. 2003, 2013; Yang et al. 2004; Fuhrmann 2012; Li et al. 2021a) successfully used marginal ripplets of *Ilyocypris* as an important taxonomic feature. Meanwhile, Mazzini et al. (2014) demonstrated that outline analysis is also useful for discriminating different species of this genus. We concur with Mazzini et al. (2014), in that outline analyses can quantify shape variation that would be difficult to characterise by observation alone.

In this study we describe a new fossil species of the genus *Ilyocypris*, based on well-preserved material from late Quaternary sediments in Inner Mongolia of China. A variety of morphological features, such as the marginal ripplets on the anterior and posterior valve margins, inner lists, valve shape, and ornamentation on the valve surface, are described and compared with those of congeners. A discussion is given about the overlap of morpho-space of the new species with some of its congeners as visualised by outline analysis, Principal Component Analysis (PCA), and cluster analysis.

## ﻿Materials and methods

### ﻿Study area and field work

**Figure 1.**
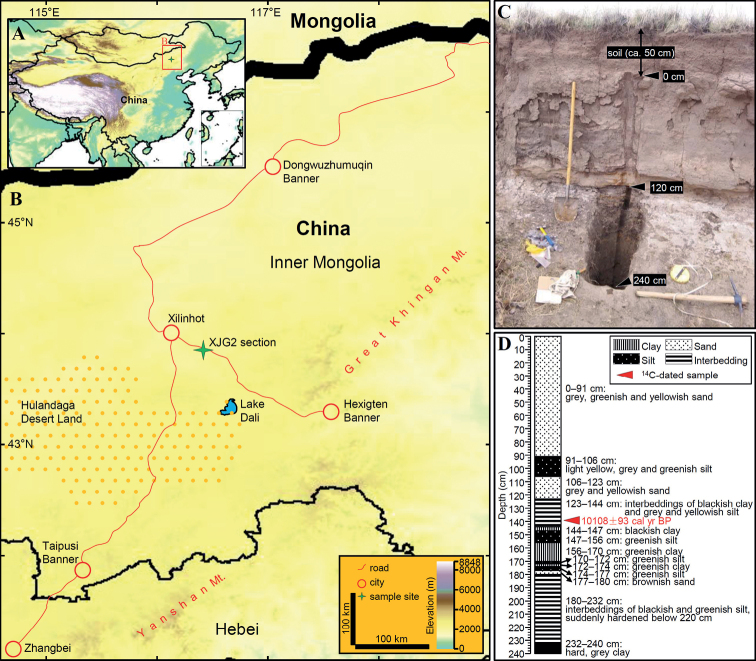
**A** digital Elevation Model showing the position of the study area (red rectangle) in central-eastern Inner Mongolia, China **B** digital Elevation Model showing the location of the XJG2 section **C** field photo of the section **D** lithology of the section. Red triangle indicates the result of ^14^C dating of gastropods from depth 139‒140 cm. See text for details.

The XJG2 section (43°51'41.9"N, 116°24'57.1"E, Fig. 1) is located c. 30 km southeast of Xilinhot City in central-eastern Inner Mongolia, China (Fig. 1). In this area, summer rainfall generates several intermittent rivers that cut into the underlying lacustrine strata. One of these rivers is named “Xiaojinggou” by local people. In May 2015, two sections were sampled on the bank of this river, XJG1 and XJG2. The section XJG2 is presented in this study.

In the field, the XJG2 section was excavated (Fig. 1C) by removing the sediment on the surface of the profile (0‒120 cm) and by digging a hole in the river bed (120‒240 cm), and in total, 240 samples were taken at 1-cm resolution. The emergence of interstitial water at a depth of 240 cm obstructed digging and sampling beneath this depth. The overlying soil, which is c. 50 cm thick and extensively interrupted with grass roots, was not sampled (Fig. 1C).

### ﻿Ostracod extraction

In the laboratory, the extraction of ostracod valves generally followed the method described by Zhai et al. (2010). Air-dried sediments were soaked overnight in 0.1% Na_2_CO_3_-buffered 10% H_2_O_2_ solution, washed through a 63-μm mesh, spread onto glass plates with a pipette and dried at room temperature. Subsequently, the ostracods were picked with a fine brush under a SZ6000 stereomicroscope. For illustration, cleaned valves were coated with gold and were imaged with a FEI Quanta 200 (Advanced Analysis and Measurement Center of Yunnan University) or a FEI Quanta 650 (Yunnan Key Laboratory for Palaeobiology, Institute of Palaeontology, Yunnan University) Scanning Electron Microscopes (SEM). All specimens from the section (Table 1) are deposited at the Yunnan Key Laboratory for Palaeobiology, Institute of Palaeontology, Yunnan University (Kunming, China).

**Table 1.** Examined valves of *Ilyocypris* from XJG2 section. All the adult left valves are identified as *Ilyocyprisleptolinea* Wang & Zhai, sp. nov. based on marginal ripplets. Others are tentatively assigned to this species. Note that all the valves of this species used for outline analysis in this study are adult left valves imaged for exterior view. Abbreviations: A, adult; A-1, last juvenile stage before adult; H, height; L, length; LV, left valve; MR, marginal ripplets; OL, outline; RV, right valve.

Specimen	Stage	RV/LV	L×H (mm)	Type designation	MR preservation	OL analysis
XJG2-021-1	A	LV	0.94×0.48	paratype	yes	yes
XJG2-136-2	A	LV	0.83×0.45	/	yes	yes
XJG2-138-2	A	LV	0.96×0.52	/	yes	/
XJG2-139-1	A	LV	0.82×0.42	/	yes	/
XJG2-153-1	A	LV	0.90×0.47	/	yes	yes
XJG2-153-2	A	LV	0.87×0.47	/	yes	yes
XJG2-153-3	A	LV	0.92×0.50	/	yes	yes
XJG2-160-1	A	LV	0.91×0.49	/	yes	/
XJG2-174-1	A	LV	0.98×0.53	/	yes	yes
XJG2-177-1	A	LV	0.90×0.47	paratype	yes	yes
XJG2-188-1	A	LV	1.01×0.53	/	yes	/
XJG2-189-1	A	LV	0.81×0.43	/	yes	yes
XJG2-190-1	A	LV	1.05×0.56	paratype	yes	yes
XJG2-192-1	A	LV	0.94×0.51	/	yes	yes
XJG2-192-2	A	LV	0.91×0.50	/	yes	yes
XJG2-192-3	A	LV	0.87×0.48	/	yes	/
XJG2-192-5	A	LV	0.78×0.43	/	yes	/
XJG2-193-1	A	LV	0.91×0.48	paratype	yes	yes
XJG2-196-4	A	LV	0.84×0.46	/	yes	/
XJG2-197-2	A	LV	0.90×0.48	paratype	yes	/
XJG2-199-2	A	LV	0.86×0.46	paratype	yes	/
XJG2-210-1	A	LV	0.94×0.48	holotype	yes	yes
XJG2-212-2	A	LV	0.94×0.52	/	yes	yes
XJG2-218-1	A	LV	0.78×0.42	/	yes	/
XJG2-228-2	A	LV	0.88×0.46	paratype	yes	yes
XJG2-192-4	A-1	LV	0.70×0.39	/	not observed due to aggregation	/
XJG2-196-1	A-1	LV	0.74×0.42	/	yes	/
XJG2-197-1	A-1	LV	0.70×0.39	/	yes	/
XJG2-199-1	A-1	LV	0.74×0.41	/	yes	/
XJG2-202-3	A-1	LV	0.80×0.44	/	yes	/
XJG2-222-1	A-1	LV	0.72×0.39	/	yes	/
XJG2-129-1	A	RV	0.91×0.47	/	/	/
XJG2-136-1	A	RV	0.93×0.50	/	/	/
XJG2-169-1	A	RV	0.87×0.48	/	/	/
XJG2-190-2	A	RV	0.90×0.49	/	/	/
XJG2-191-1	A	RV	0.83×0.45	/	/	/
XJG2-193-2	A	RV	0.90×0.47	/	/	/
XJG2-196-2	A	RV	0.88×0.47	/	/	/
XJG2-196-3	A	RV	0.95×0.50	/	/	/
XJG2-197-5	A	RV	0.83×0.46	/	/	/
XJG2-197-6	A	RV	0.90×0.49	/	/	/
XJG2-202-1	A	RV	0.86×0.46	/	/	/
XJG2-202-4	A	RV	0.85×0.45	/	/	/
XJG2-203-1	A	RV	0.85×0.45	/	/	/
XJG2-203-2	A	RV	0.86×0.45	/	/	/
XJG2-205-2	A	RV	0.85×0.45	/	/	/
XJG2-212-3	A	RV	0.82×0.44	/	/	/
XJG2-216-1	A	RV	0.99×0.53	/	/	/
XJG2-217-1	A	RV	0.86×0.47	/	/	/
XJG2-218-2	A	RV	0.87×0.47	/	/	/
XJG2-221-1	A	RV	0.81×0.45	/	/	/
XJG2-226-1	A	RV	0.88×0.47	/	/	/
XJG2-228-3	A	RV	0.79×0.43	/	/	/
XJG2-192-6	A-1	RV	0.77×0.42	/	/	/

### ﻿Sample dating

In the sample XJG2-140 (depth 139‒140 cm), a small number of well-preserved gastropods were found, which were used for ^14^C dating.

Pre-treatment of the dating material was done in the Laboratory Test Center, Institute of Geology, China Earthquake Administration (Beijing, China). Brush-cleaned gastropod shells were soaked in 0.1N HCl in an ultra-sonic bath until the surface layer (c. 1/3 in thickness) was eroded away. The residual was rinsed with deionised water and was then oven-dried. Approximately 20 mg of such cleaned, dried shell was dissolved with 1 ml of H_3_PO_4_ at 70 °C in an airtight reacting system with a pressure of ~ 10^-2^ Pa. The released CO_2_ was purified and was deoxidised by H_2_, with Fe powder as the catalyst, yielding ~ 1 mg of graphite, which was dated in a NEC 1.5SDH-1 Compact PKUAMS facility at the Institute of Heavy Ion Physics, Peking University (Beijing, China). The ^14^C age of the sample was calibrated using the OxCal v. 4.2 program (Bronk Ramsey 2009).

### ﻿Numerical analyses

In order to characterise and then to compare the shapes of different valves, outline analysis was performed, with general principles following those provided by Baltanás and Danielopol (2011). First, each SEM image of the valve was digitised with the software tpsDig2 (v. 2.31) (Rohlf 2017) to obtain coordinates of the outline. Subsequently, the outline was located (with the geometric centre set at (0, 0)), rotated and flipped if necessary (to obtain the same orientations for exterior and interior views and for left and right valves), and scaled (standardised for the equal surface area of 5000). Afterwards, two different approaches were used to calculate the dissimilarity between the outlines to produce dissimilarity matrices. In the first approach, all the outlines were fixed, i.e., with the antero-ventral and the postero-ventral parts rested on a horizontal axis (cf. Meisch 2000: fig. 3D), and the dissimilarity (Fixed Outline Canberra Dissimilarity, FOCD) was calculated. The second approach allows rotation of one of the two outlines under comparison by ± 20 degrees, and the smallest dissimilarity value, which is called the Minimal Running Canberra Dissimilarity (MRCD) index herein, was used. This approach was to avoid unreasonable superimposing of the length axis that may have been altered by subtle variations of the shapes of the anterior and the posterior ends of the valve. The dissimilarity matrices, resulted from the two different approaches, were used respectively for cluster analysis, which employed a group-average strategy (Sokal and Michener 1958). All the computation and illustration of the outline coordinates were made with the software Microsoft Office Excel 2019 and Microsoft Office PowerPoint 2019.

Principal Component Analysis (PCA) was performed on the polar-coordinate data of the fixed outlines (see above) using the software CANOCO v. 4.5 (Ter Braak and Šmilauer 2002), and biplots of the first two axes of PCA were used to compare the morpho-spaces of different species.

A total of 42 outlines of the valves of *Ilyocypris* were analysed (Tables 1, 2). Apart from the *Ilyocypris* material from the XJG2 section, four other congeneric species with Holocene representatives in eastern Inner Mongolia were included: *I.bradyi*, *Ilyocyprisinnermongolica* Zhai & Xiao, 2013, *Ilyocyprismongolica* Martens, 1991, and *I.japonica*. The first three species have been found living in eastern Inner Mongolia (Zhai and Xiao 2013; Zhai and Zhao 2014; Zhai et al. 2022). A marginal-ripplet pattern coinciding with that of *I.japonica* was found by DZ in the *Ilyocypris* material from Holocene sediments of Lake Hulun in northeastern Inner Mongolia, which had been left in open nomenclature in Zhai et al. (2011). In view of the paucity of intact valves from Inner Mongolia, however, images of Quaternary material of *I.bradyi* from UK (including six specimens from DJH’s collection and two from the Natural History Museum (London, UK) collection, the latter published in Whittaker and Parfitt 2017) and images of extant material of *I.japonica* from Japan were utilised (Table 2). The exterior views of left valves provided by the Scanning Electron Microscope (SEM) were used, with the following exceptions. First, for *I.innermongolica*, only exterior views of left valves and exterior and interior views of right valves of the type specimens were available due to past experimental design. The single specimen of *I.bradyi* from the study area was used with only the exterior view of the right valve because the left valve had been damaged during dissection.

**Table 2.** Examined valves of other species of *Ilyocypris*. All the specimens are adults, which can be judged from their larger sizes, wider calcified inner lamellae, and patterns of inner marginal ripplets (only applied to left valves). Additional abbreviations to those in Table 1: ext, exterior view; int, interior view; MIS, marine isotopic stage.

Species	Specimen number	RV/LV, int/ext	L×H (mm)	Locality	Horizon	OL analysis
*I.bradyi*	CC22	LV, ext	0.85×0.44	Greenlands Pit, Purfleet, UK	MIS 9 (Pleistocene)	yes
*I.bradyi*	CC23	LV, ext	0.86×0.44	Greenlands Pit, Purfleet, UK	MIS 9 (Pleistocene)	yes
*I.bradyi*	CC26	LV, ext	0.85×0.43	Greenlands Pit, Purfleet, UK.	MIS 9 (Pleistocene)	yes
*I.bradyi*	MTDB-3	LV, ext	1.06×0.55	Marks Tey, UK	MIS 11 (Pleistocene)	yes
*I.bradyi*	MTDB-5	LV, ext	0.98×0.51	Marks Tey, UK	MIS 11 (Pleistocene)	yes
*I.bradyi*	MTDB-13	LV, ext	1.04×0.55	Marks Tey, UK	MIS 11 (Pleistocene)	yes
*I.bradyi*	NHM UK PM OS 19853	LV, ext	no data	Boxgrove, UK (cf. Whittaker and Parfit 2017)	MIS 13 (Pleistocene)	yes
*I.bradyi*	NHM UK PM OS 19854	LV, ext	no data	Boxgrove, UK (cf. Whittaker and Parfit 2017)	MIS 13 (Pleistocene)	yes
*I.bradyi*	dyzoc810	RV, ext	0.83×0.43	Creek Y16, Inner Mongolia, China (cf. Zhai et al. 2022)	extant	yes
*I.mongolica*	dyzoc515	LV, ext	0.80×0.43	Lake X26, Inner Mongolia, China (cf. Zhai et al. 2022)	extant	yes
*I.mongolica*	dyzoc518	LV, ext	0.89×0.48	Lake X26, Inner Mongolia, China (cf. Zhai et al. 2022)	extant	yes
*I.mongolica*	LBM 1430009163	LV, ext	0.83×0.46	Pond X36, Inner Mongolia, China (cf. Zhai and Zhao 2014)	extant	yes
*I.innermongolica*	dyzoc5	LV, int	0.73×0.37	Lake Hulun, China (cf. Zhai and Xiao 2013)	extant	yes
*I.innermongolica*	dyzoc5	RV, ext	0.71×0.36	Lake Hulun, China (cf. Zhai and Xiao 2013)	extant	yes
*I.innermongolica*	dyzoc7	LV, int	0.76×0.42	Lake Hulun, China (cf. Zhai and Xiao 2013)	extant	yes
*I.innermongolica*	dyzoc7	RV, ext	0.74×0.39	Lake Hulun, China (cf. Zhai and Xiao 2013)	extant	yes
*I.innermongolica*	dyzoc8	LV, int	0.69×0.37	Lake Hulun, China (cf. Zhai and Xiao 2013)	extant	yes
*I.innermongolica*	dyzoc8	RV, ext	0.69×0.36	Lake Hulun, China (cf. Zhai and Xiao 2013)	extant	yes
*I.innermongolica*	dyzoc93	LV, int	0.70×0.36	Lake Hulun, China (cf. Zhai and Xiao 2013)	extant	yes
*I.innermongolica*	dyzoc93	RV, int	0.67×0.35	Lake Hulun, China (cf. Zhai and Xiao 2013)	extant	yes
*I.innermongolica*	dyzoc100	LV, int	0.74×0.37	Lake Hulun, China (cf. Zhai and Xiao 2013)	extant	yes
*I.innermongolica*	dyzoc100	RV, ext	0.73×0.36	Lake Hulun, China (cf. Zhai and Xiao 2013)	extant	yes
*I.innermongolica*	dyzoc114	LV, int	0.73×0.37	Lake Hulun, China (cf. Zhai and Xiao 2013)	extant	yes
*I.innermongolica*	dyzoc114	RV, ext	0.71×0.36	Lake Hulun, China (cf. Zhai and Xiao 2013)	extant	yes
*I.japonica*	LBM 1430009143	LV, ext	0.91×0.50	Shiga Prefecture, Japan (cf. Smith et al. 2019)	extant	yes
*I.japonica*	LBM 1430009146	LV, ext	0.76×0.40	Gyeongsangnam-do, South Korea (cf. Smith et al. 2019)	extant	yes
*I.japonica*	LBM 1430009147	LV, ext	0.86×0.46	Gyeongsangnam-do, South Korea (cf. Smith et al. 2019)	extant	yes

## ﻿Results

### ﻿Lithology and chronology

The 240-cm thick lacustrine XJG2 section consists of clay, silt, and fine-sand fractions and contains no gravels (Fig. 1D). No obvious sedimentary hiatus is detected within the section. Its upper 123 cm is dominated by fine to medium sand but is interrupted by silt at 91‒106 cm. The interval of 123‒144 cm contains interbeds of clay and silt. The depths from 144 to 180 cm are represented by frequent alternations of clay, silt, and sand layers. Interbeds of silt bands with different colours occur in 180‒232 cm. The bottom part of the section, 232‒240 cm, consists of hard grey clay.

The gastropod shells (laboratory dating code: CG-2021-1566) from the sample XJG2-140 in the middle part of the XJG2 section (Fig. 1D) yielded a ^14^C age of 8960 ± 30 yr that is calibrated to 10108 ± 93 cal yr BP (calendar years before the present; present = 1950 AD). This is close to the beginning of the Holocene Epoch, 11700 cal yr BP, based on the International Chronostratigraphic Chart v. 2022/10 (Cohen et al. 2013, updated). Considering the reported sedimentation rates of lakes in eastern Inner Mongolia, which varied from 0.15 m/kyr (Lake Hulun, Zhai et al. 2011) to 1.1 m/kyr (Lake Daihai, Xiao et al. 2004), the age of the bottom part of the XJG2 section probably extends into the late Pleistocene. Therefore, the age of this section should belong to the late Quaternary.

### ﻿Systematics

In addition to *Ilyocypris*, the fossil ostracods recovered from the XJG2 section include members of the genera *Candona* Baird, 1845, *Cypridopsis* Brady, 1867, *Fabaeformiscandona* Krstić, 1972 and *Pseudocandona* Kaufmann, 1900 (work in progress, to be published elsewhere). Valves of *Ilyocypris* were found between the depths of 240 cm and 20 cm. Examination of the morphologies of the left valves, including shape, ornamentation, and marginal ripplets, suggests that they belong to a new species, which is described below.

### ﻿Class Ostracoda Latreille, 1802

**Order Podocopida Sars, 1866**

**Superfamily Cypridoidea Baird, 1845**

**Family Ilyocyprididae Kaufmann, 1900**

**Subfamily Ilyocypridinae Kaufmann, 1900**

**Genus *Ilyocypris* Brady & Norman, 1889**

#### ﻿ Ilyocypris leptolinea

Taxon classificationAnimaliaPodocopidaIlyocyprididae

Wang & Zhai sp. nov.

787199DE-2040-516B-BA5C-6C967F16833B

https://zoobank.org/A551E7F9-DD93-4A16-9557-2ED465EA4221

Figs 2 , 3 , 4

##### Type locality.

XJG2 section (43°51'41.9"N, 116°24'57.1"E, Fig. 1), lacustrine outcrop cut by intermittent river in central-eastern Inner Mongolia, China.

##### Type horizon.

Late Pleistocene to Holocene; holotype from 209‒210 cm depth in section, c. 70 cm below level of gastropod shells that yielded a calibrated radiocarbon date of 10,108 ± 93 calendar years before present (cal yr BP).

##### Type material.

**Figure 2.**
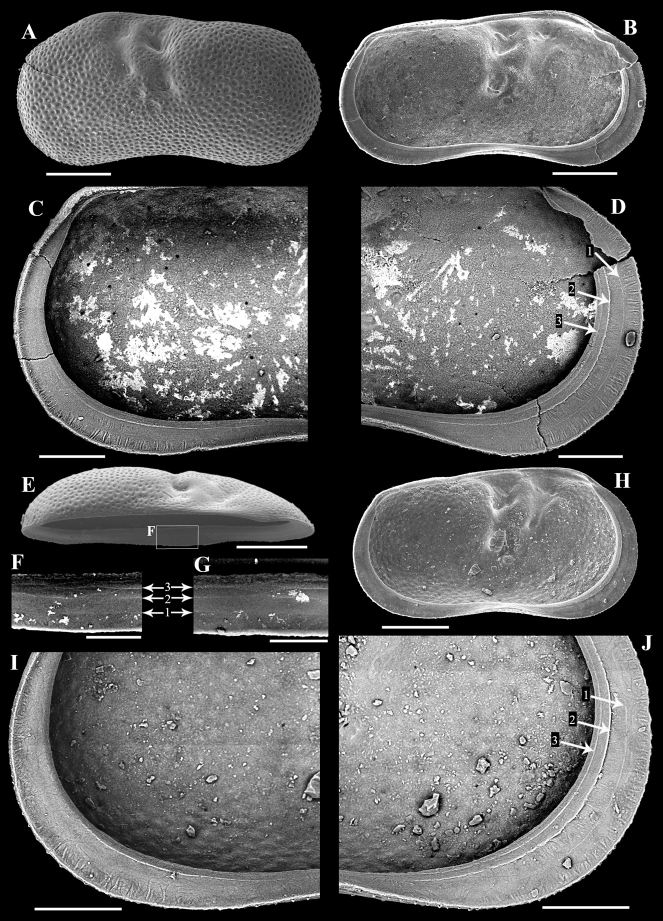
Adult left valves of the ostracod *Ilyocyprisleptolinea* Wang & Zhai, sp. nov. from the late Quaternary Xiaojinggou section of Inner Mongolia, China **A–D** XJG2-210-1 (holotype, 0.94 × 0.48 mm) **A** exterior view **B** interior view **C** interior view of the posterior part, showing the marginal ripplets **D** interior view of the anterior part, showing the marginal ripplets. Arrows and numerals indicate three inner lists on the calcified inner lamella **E, F** XJG2-206-4 **E** oblique-dorsal view **F** enlarged view of the rectangle in (E), showing the crossing pattern of the inner lists at the median part of ventral margin. Arrows and numerals indicate the three inner lists **G** XJG2-206-1, enlarged oblique-dorsal view of the median part of calcified inner lamella, showing the crossing pattern of inner lists **H–J** XJG2-199-2 (paratype) **H** interior view **I** interior view of the postero-ventral part, showing the marginal ripplets **J** Interior view of the antero-ventral part, showing the marginal ripplets. Arrows and numerals indicate the three inner lists. Scale bars: 200 μm (**A, B, E, H**); 100 μm (**C, D, I, J**); 50 μm (**F, G**).

**Figure 3.**
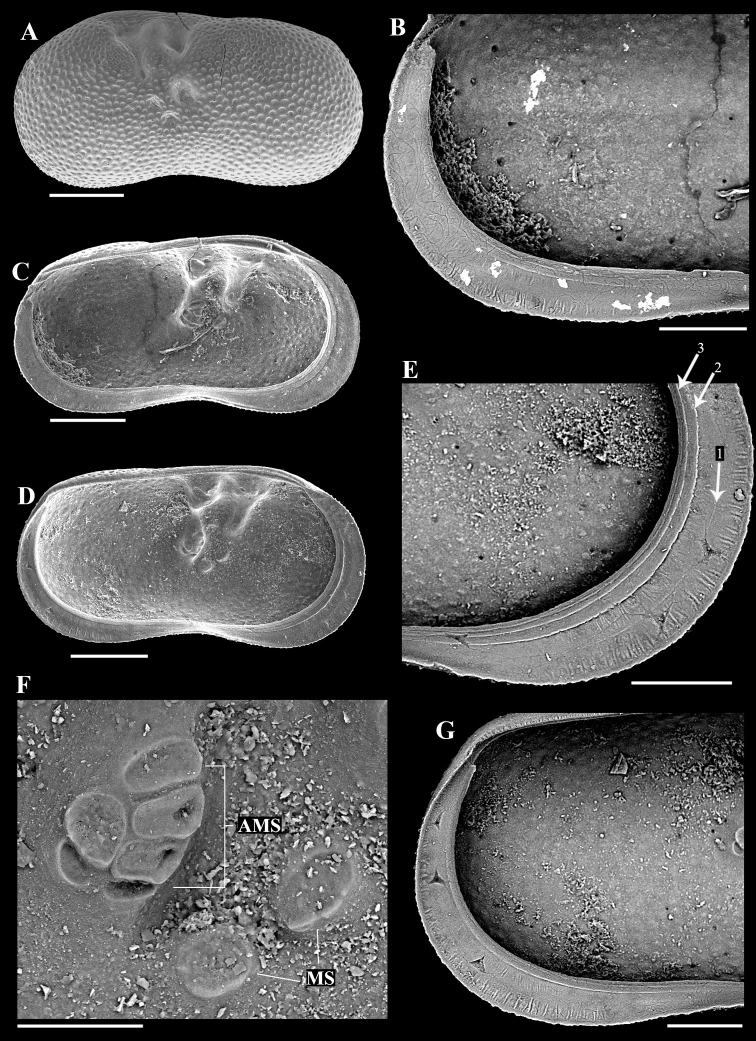
Adult left valves of the ostracod *Ilyocyprisleptolinea* Wang & Zhai, sp. nov. from the late Quaternary Xiaojinggou section of Inner Mongolia, China **A–C** XJG2-21-1 (paratype) **A** exterior view **B** interior view of the postero-ventral part, showing the marginal ripplets **C** interior view **D–G** XJG2-197-2 (paratype) **D** interior view **E** interior view of the antero-ventral part, showing the marginal ripplets and three inner lists (arrowed) **F** adductor muscle scars (AMS) and mandibular scars (MS) **G** interior view of the posterior part, showing the marginal ripplets. Scale bars: 200 μm (**A, C, D**); 100 μm (**B, E, G**); 50 μm (**F**).

***Holotype***: adult left valve, XJG2-210-1 (XJG2, section code; 210, sample code, corresponding to 209‒210 cm depth in section; 1, registration number), length 0.94 mm, height 0.48 mm. ***Paratypes***: seven adult left valves, XJG2-021-1, XJG2-177-1, XJG2-190-1, XJG2-193-1, XJG2-197-2, XJG2-199-2, XJG2-228-2. All type specimens with marginal ripplets on anterior and posterior calcified inner lamellae well-preserved (Figs 2, 3).

##### Other material examined.

**Figure 4.**
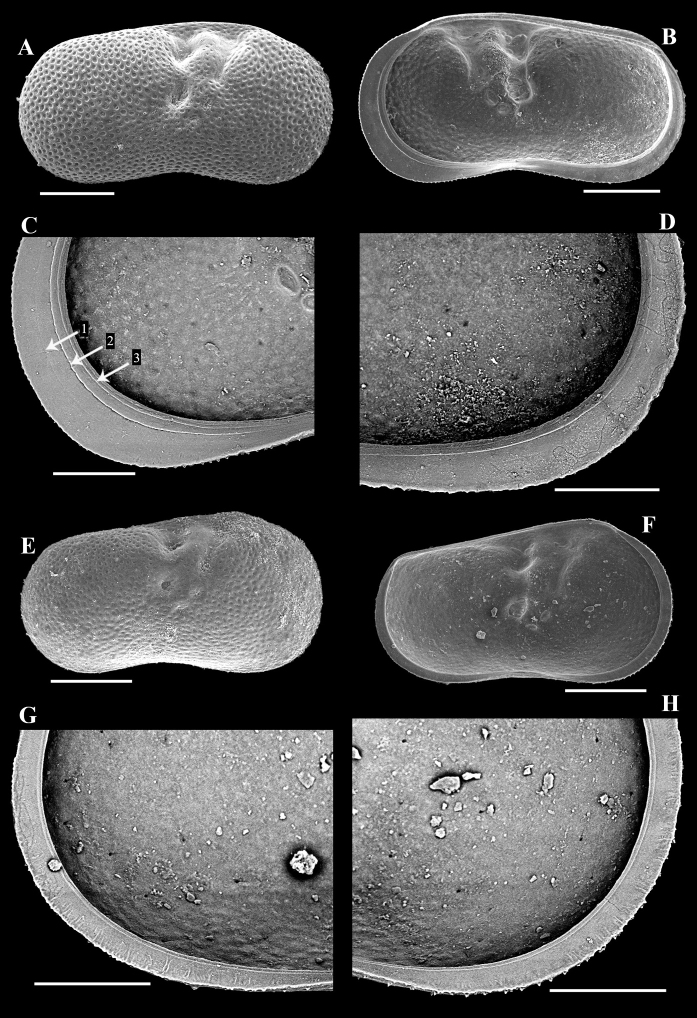
Ostracod valves tentatively identified as *Ilyocyprisleptolinea* Wang & Zhai, sp. nov. from the late Quaternary Xiaojinggou section of Inner Mongolia, China **A–D** XJG2-203-1, adult right valve **A** exterior view **B** interior view **C** interior view of the antero-ventral part, showing three inner lists (arrowed) **D** interior view of the postero-ventral part **E** XJG2-193-4, right valve of A-1 juvenile, exterior view **F–H** XJG2-199-1, left valve of A-1 juvenile **F** interior view **G** interior view of the postero-ventral part, showing the marginal ripplets **H** interior view of the antero-ventral part, showing the marginal ripplets. Scale bars: 200 μm (**A, B, E, F**); 100 μm (**C, D, G, H**).

**Figure 5.**
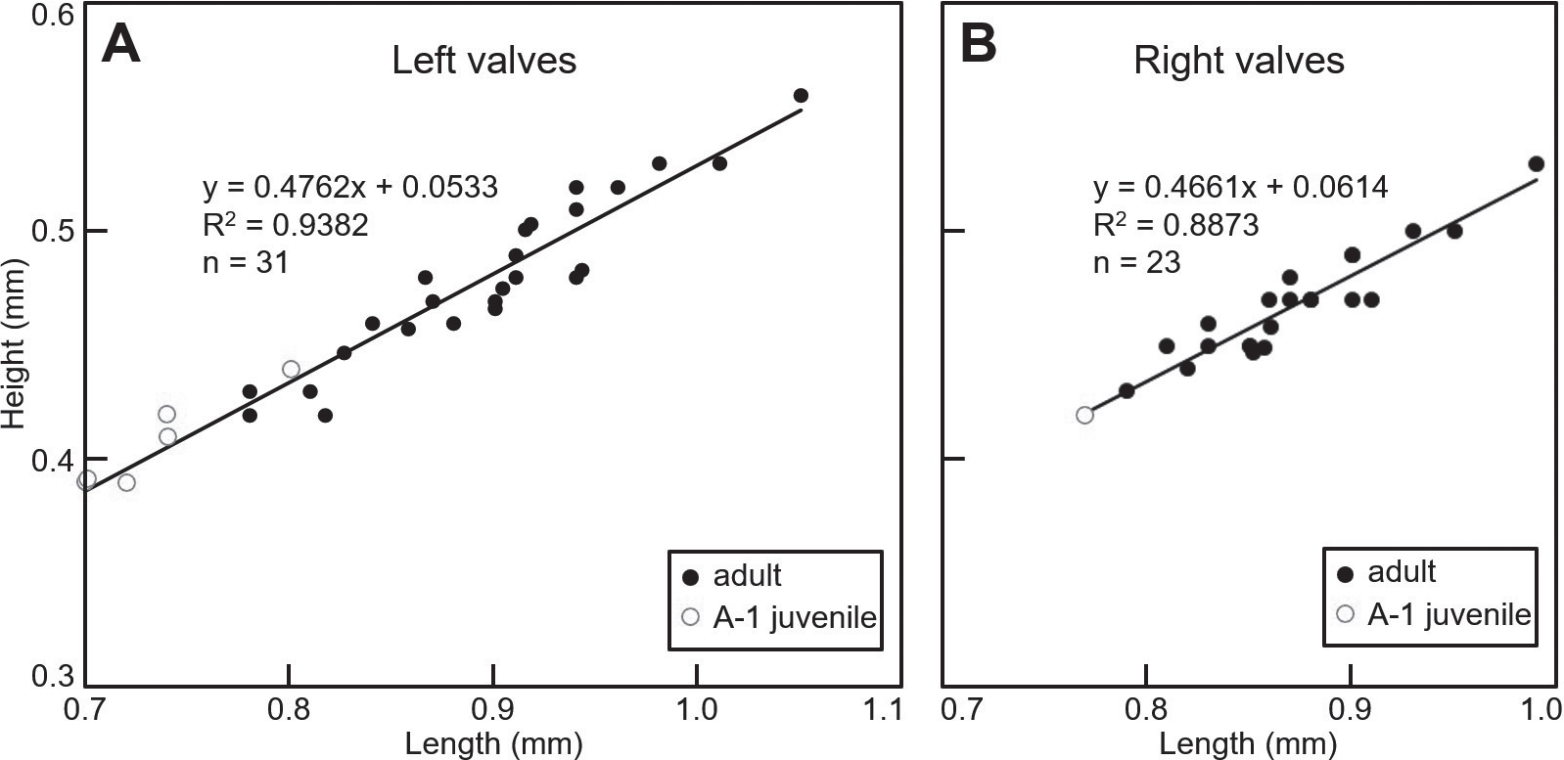
Height‒length biplots of the left valves of *Ilyocyprisleptolinea* Wang & Zhai, sp. nov. (**A**) and the right valves tentatively identified as this species (**B**) from the late Quaternary Xiaojinggou section of Inner Mongolia, China.

17 adult left valves, 22 adult right valves, six A-1 juvenile left valves, and one A-1 juvenile right valve (Table 1; Figs 2–5). Adult right valves and juvenile valves provisionally identified as this species in view of monospecific adult left valves in section.

##### Etymology.

From the Greek *leptos* for fine, small, or subtle, and Latin *linea* for line or thread, referring to fine marginal ripplets on calcified inner lamellae of LV.

##### Dimensions.

Adult left valves (*n* = 25, Fig. 5A) with length 0.78‒1.05 mm, height 0.42‒0.56 mm, H/L ratio 0.51‒0.55. Adult right valves (*n* = 22, Fig. 5B) with length 0.79‒0.99 mm, height 0.43‒0.53 mm, H/L ratio 0.52‒0.56. Juvenile valves slightly smaller but with size ranges overlapping with those of adult valves (Fig. 5).

##### Diagnosis.

Intermediate-sized *Ilyocypris* (length ranging from 0.78 to 1.05 mm, Table 1) with shape, sulci, and pits typical of genus. Valve surface without nodes, occasionally with tiny spines along anterior and/or posterior margins. Calcified inner lamellae wide, bearing (in left valve only) two rows of densely arranged, fine marginal ripplets along entire anterior and posterior valve margins; distal row near valve margin, usually well expressed; proximal row in intermediate area less pronounced, absent in poorly preserved specimens. One inner list present between two rows of marginal ripplets in well-preserved specimens.

##### Description.

***Left valves*.** Intermediate-sized *Ilyocypris*. Valve sub-reniform in lateral view, with greatest height (antero-dorsal corner) at anterior third. Dorsal margin, i.e., section between antero-dorsal and postero-dorsal corners, nearly straight but with blunt turn immediately behind posterior sulcus due to inflation of postero-dorsal part. Anterior margin broadly rounded, with dorsal part nearly straight and more ventral parts evenly rounded. Posterior margin evenly rounded and less obtrusive. Ventral margin concave. Valve surface carrying two transverse sulci, with anterior sulcus originating from antero-dorsal corner, tapering ventrally, and terminating slightly above mid-height. Posterior sulcus wider and shorter. Adductor muscle scars situated in ovate depression immediately below posterior sulcus. Mandibular scars situated in two small depressions to antero-ventral position of adductor muscle scars. Shell surface densely covered with small pits, with those in front of anterior sulcus, between two sulci and behind posterior sulcus, smaller. Small number of tiny spines present along anterior and posterior margins in some specimens.

Interior view, anterior and posterior calcified inner lamellae comparatively wide but with anterior one slightly wider. Three inner lists present on anterior calcified inner lamella (Figs 2D, J, 3E). First one running in intermediate zone, usually weakly expressed, sometimes not preserved. Second and third ones running close to inner margin. Inner lists also present on posterior calcified inner lamella but with first one usually very faint or absent (Figs 2C, I, 3B, G). Two rows of fine, densely arranged marginal ripplets present on both anterior and posterior calcified inner lamellae (Figs 2, 3): distal row (Figs 2C, D, I, J, 3B, E, G) with each ripplet extending from exterior margin of selvage near valve margin to almost first inner list, distributed throughout entire anterior and posterior calcified inner lamellae, usually well expressed; proximal zone (Figs 2C, D, J, 3B, E, G) present between first and second inner lists, better observed at antero-ventral and postero-ventral areas, sometimes not preserved (Fig. 2I). Adductor muscle scars (Fig. 3F), consisting of six scars of different sizes and shapes, arranged in two rows (four anterior scars and two posterior ones). Mandibular scars (Fig. 3F) consisting of two sub-oval elements.

Dorsal view (Fig. 2E) semi-elliptical, with part behind posterior sulcus wider than anterior part. Anterior end pointed. Posterior end bluntly pointed. Antero-dorsal corner with one small blunt expansion representing tooth-structure of hinge. Middle part of ventral calcified inner lamella (corresponding to highest part of ventral margin in lateral view; observed from oblique-dorsal view) with gentle, inward, i.e., adaxial, expansion. Two proximal inner lists, i.e., second and third ones, showing complex crossing patterns on this part (Fig. 2F, G).

***Right valves*.** Shape similar to that of left valve, but dorsal margin straighter (Fig. 4A, B) with posterior section not inflated dorsally (Fig. 4A, B). Valve margin also with three inner lists but first one very faint on anterior calcified inner lamella (Fig. 4C) and almost absent on posterior calcified inner lamella (Fig. 4D).

***Valves of A*-*1 juveniles*.** Shape similar to that of adults but with dorsal margin more inclined (Fig. 4E, F). Pits on valve surface smaller and shallower. Calcified inner lamellae of left valve narrow, with only one row of densely arranged marginal ripplets distally: extending to median area in anterior calcified inner lamella (Fig. 4H), almost to proximal area in postero-ventral area (Fig. 4G). Only one inner list present, running close to inner margin (Fig. 4G, H).

##### Differential diagnosis.

The new species can be easily distinguished from congeners by the fineness, number, and distribution of the two rows of marginal ripplets as well as the presence of the outer-most (first) inner list in the intermediate zone that separates the two rows of marginal ripplets. *Ilyocyprisbradyi*, for example, has fewer, thicker, and more widely spaced ripplets confined to the postero-ventral area, typically four or five in the outer row (see e.g., Mazzini et al. 2014: fig. 11). Considering potential taphonomic loss of finer morphologies in fossil material (e.g., the abrasion of the proximal row of marginal ripplets and the inner lists), several species with morphologies that may be confused with the poorly preserved specimens of the new species are compared here. *Ilyocyprislacustris* Kaufmann, 1900 seems to have two rows of fine marginal ripplets separated by one inner list on the anterior valve margin, which resembles the new species (Fuhrmann 2012: Tafel (= pl.) 76 1b), but the ripplets on the posterior part are confined to the postero-ventral margin and consist of only one row (Fuhrmann 2012: Tafel (= pl.) 76 1f and 2d). The highest point of the carapace of *I.lacustris* is situated at approximately the anterior quarter, more anterior than that of the new species. The marginal ripplets on the posterior part of *I.salebrosa* are also fine and consist of two rows (Mazzini et al. 2014: fig. 11, panel 8), but are confined to the postero-ventral part too. And the prominent postero-dorsal node on the exterior valve surface offers easy distinction from the new species. *Ilyocyprishanguk* Karanovic & Lee, 2013 described from South Korea, has a valve shape somewhat similar to the new species, and its H/L ratio (0.55 for LV and 0.54 for RV as measured from the holotype in Karanovic and Lee 2013: fig. 6A, B) comes close to that of the new species. However, no marginal ripplets were observed on the left valve of *I.hanguk* (Karanovic and Lee 2013: fig. 6A, D). Furthermore, small lateral projections are present on the postero-central part of the valve of *I.hanguk* (Karanovic and Lee 2013: fig. 6B), which are not observed in *I.leptolinea* Wang & Zhai, sp. nov. (Figs 2–4). *Ilyocyprisglabella* Fuhrmann & Goth, 2011, *Ilyocyprissebeiensis* Yang & Sun, 2004 (in Yang et al. 2004), *I.tibeta* (Peng et al. 2021), and *Qinghaicypriscrassa* Huang, 1979 (Yang et al. 2004) possess marginal ripplets distributed along most parts of the anterior and posterior calcified inner lamellae. However, in all these species, there is only one row of ripplets distributed near the valve margins, which are thicker and more sparsely arranged compared with the new species. As a result, even if the proximal row of marginal ripplets is taphonomically lost in the new species, it would not be confused with these species. Besides, the valves of *I.glabella*, *I.sebeiensis*, and *Q.crassa* are significantly stouter than those of the new species. (Note that Shen et al. (1993) and Hou et al. (2002) considered *Qinghaicypris* a junior synonym of *Ilyocypris* and moved all the species in this genus to *Ilyocypris*).

**Figure 6.**
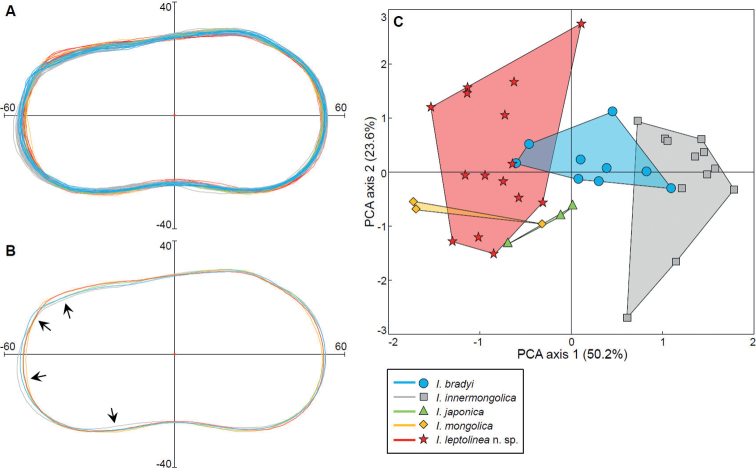
Illustrations showing the overlap of the outline morpho-spaces of five species of *Ilyocypris* that have living or Quaternary representatives in eastern Inner Mongolia. All outlines have been standardised for equal surface area, rotated with the antero-ventral and the postero-ventral parts resting horizontally, and with the geometric centres set at (0, 0) **A** outlines of 42 valves **B** mean outlines of the five species. Arrows indicate areas showing relatively large inter-species variability, i.e., the postero-dorsal‒posterior and the postero-ventral areas **C** principal Component Analysis (PCA) biplot of 42 valves based on their outline data. Coloured shaded areas indicate the morpho-spaces of the five species, cast on the two-dimensional space defined by PCA axis 1 and 2.

### ﻿Valve outlines of different species of *Ilyocypris*

The dissimilarity matrices of the specimens (Tables 3, 4) summarise the intra- and inter-species variabilities of the outlines of different *Ilyocypris* species. Although the intra-species dissimilarity values are generally small (FOCD of 0.61‒1.00 and MRCD of 0.61‒0.96, respectively), they show considerable overlap with the range of the inter-species dissimilarity values among the species analysed (FOCD of 0.80‒1.58 and MRCD of 0.72‒1.42, respectively) (Tables 3, 4). In particular, *I.leptolinea* Wang & Zhai, sp. nov. exhibits considerable intra-species shape variation (e.g., FOCD of 1.00, Table 3) that is comparable to its inter-species difference with *I.bradyi*, *I.japonica*, and *I.mongolica* (Table 3). Correspondingly, the superimposition of the outlines of different specimens reveals considerable within-species variation (Fig. 6A) while comparatively small between-species difference (Fig. 6B). Although these species show some shape difference on the postero-dorsal, posterior, and postero-ventral valve areas (Fig. 6B), such disparities are small. In fact, the morpho-space of *I.leptolinea* Wang & Zhai, sp. nov. comes very close to, or even overlaps with those of *I.bradyi*, *I.japonica*, and *I.mongolica*, although separated from that of *I.innermongolica* (Fig. 6C).

**Table 3.** Average intra- (**bold**) and inter-species outline dissimilarity of the five species of *Ilyocypris* analysed in this study, based on a Fixed Outline Canberra Dissimilarity (FOCD, %) index between the specimens analysed in this study. Small numerals in parentheses indicate the number of dissimilarity values used for averaging. Species abbreviations: *Ibr*, *I.bradyi*; *Iep*, *I.leptolinea* Wang & Zhai, sp. nov.; *Iin*, *I.innermongolica*; *Ija*, *I.japonica*; *Imo*, *I.mongolica*.

Species	*Iep*	*Ibr*	*Imo*	*Iin*	*Ija*
*Iep*	**1.00_(105)_**				
*Ibr*	1.18_(135)_	**0.84_(36)_**			
*Imo*	1.01_(45)_	1.21_(27)_	**0.81_(3)_**		
*Iin*	1.49_(180)_	1.14_(108)_	1.58_(36)_	**0.83_(66)_**	
*Ija*	1.00_(45)_	0.92_(27)_	0.80_(9)_	1.17_(36)_	**0.61_(3)_**

**Table 4.** Average intra- (**bold**) and inter-species outline dissimilarity of the five species of *Ilyocypris* analysed in this study, based on a Minimal Running Canberra Dissimilarity (MRCD, %) index. Small numerals in parentheses indicate the number of dissimilarity values used for averaging. Species abbreviations: *Ibr*, *I.bradyi*; *Iep*, *I.leptolinea* Wang & Zhai, sp. nov.; *Iin*, *I.innermongolica*; *Ija*, *I.japonica*; *Imo*, *I.mongolica*.

Species	*Iep*	*Ibr*	*Imo*	*Iin*	*Ija*
*Iep*	**0.96_(105)_**				
*Ibr*	1.10_(135)_	**0.76_(36)_**			
*Imo*	0.95_(45)_	1.10_(27)_	**0.70_(3)_**		
*Iin*	1.38_(180)_	1.07_(108)_	1.42_(36)_	**0.71_(66)_**	
*Ija*	0.93_(45)_	0.87_(27)_	0.72_(9)_	1.12_(36)_	**0.61_(3)_**

**Figure 7.**
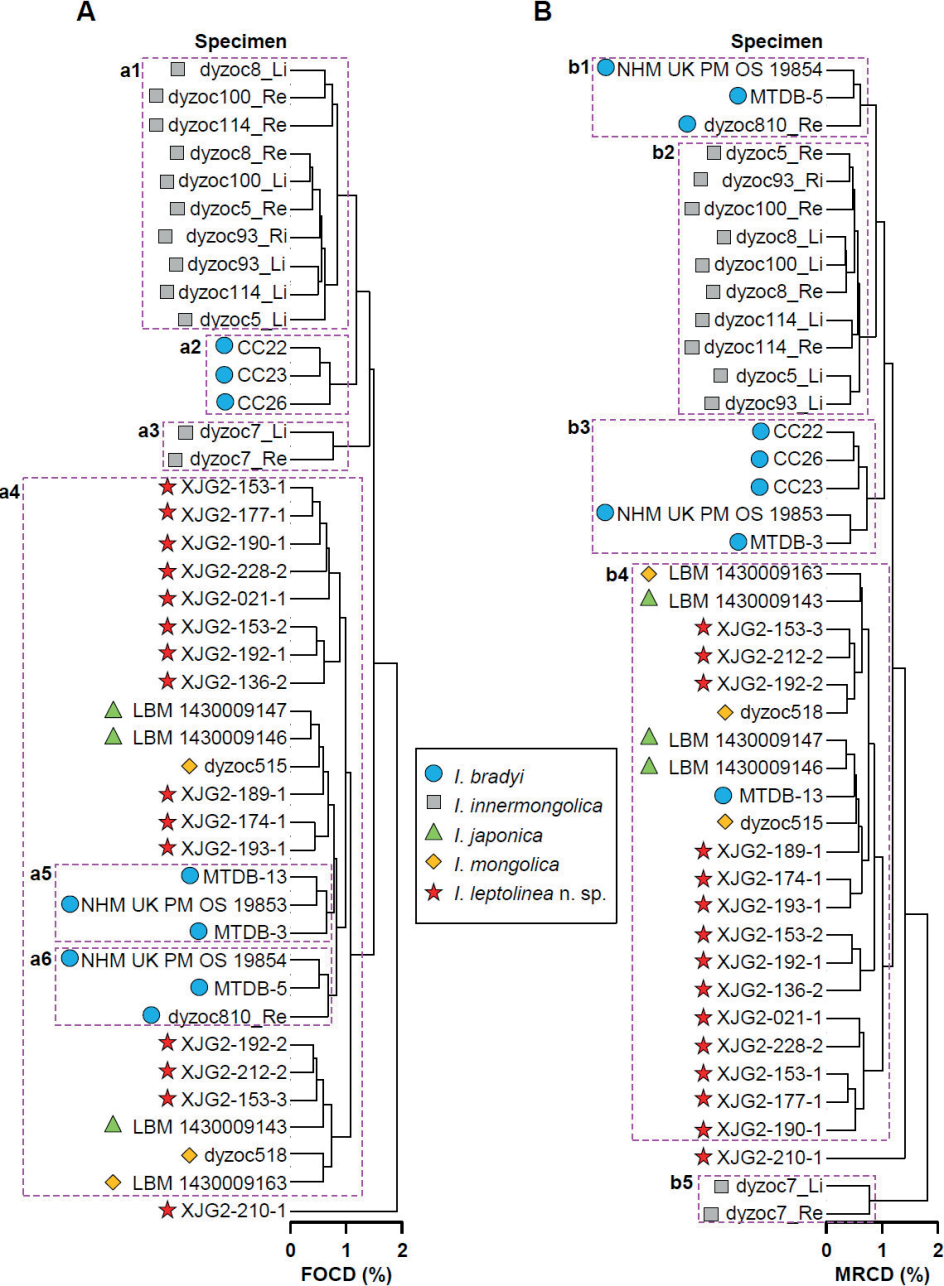
Cluster dendrograms of the valve outlines of *Ilyocypris* species based on the matrices of ‘Fixed Outline Canberra Dissimilarity’ (FOCD, with antero-ventral and postero-ventral parts of valves placed horizontally) (**A**) and ‘Minimal Running Canberra Dissimilarity’ (MRCD) (**B**) showing overlap of the morpho-spaces between congeners. The analysed *Ilyocypris* species are those with living or Quaternary representatives in eastern Inner Mongolia. Pink dashed rectangles indicate groups of specimens mentioned in the text. See text for details.

In the cluster analyses based on the two dissimilarity indices (Fig. 7A, B), *I.leptolinea* Wang & Zhai, sp. nov. exhibits a ‘mixed’ pattern with *I.bradyi*, *I.japonica*, and *I.mongolica*, albeit separated from *I.innermongolica*. In both cluster dendrograms, most of the specimens of *I.leptolinea* Wang & Zhai, sp. nov. are distributed in small clusters encompassed by the big cluster (a4 or b4 in Fig. 7) that also contains *I.bradyi*, *I.japonica*, and *I.mongolica*. Among all the species, *I.innermongolica* is the only one that can be readily distinguished from others by valve outline alone (clusters a1 and a3; b2 and b5 in Fig. 7), although its various specimens do not form a single clade (Fig. 7A, B). Most of the specimens of *I.bradyi* may also be recognised by valve outline (clusters a2, a5 and a6; b1 and b3 in Fig. 7), but these small clusters are embedded in the same cluster with either *I.leptolinea* Wang & Zhai, sp. nov., *I.japonica*, and *I.mongolica* (clusters a5 and a6 in Fig. 7A) or with *I.innermongolica* (cluster a2 in Fig. 7A and clusters b1 and b3 in Fig. 7B).

The above-mentioned partial-separation pattern of *Ilyocypris* species in the cluster dendrograms (Fig. 7) is in correspondence with the similar valve shapes of these species (Fig. 6B), as well as the intra-species shape plasticity (Fig. 6C).

## ﻿Discussion

### ﻿Taxonomy of the valve material of *Ilyocypris*

Based on the morphologies of valve material, we have herein erected *Ilyocyprisleptolinea* Wang & Zhai, sp. nov. from late Quaternary lacustrine strata of Inner Mongolia. This species is best recognised by its two rows of densely arranged fine inner marginal ripplets on both the anterior and the posterior calcified inner lamellae of the left valve. Other features, such as the three inner lists, the relatively great width of the calcified inner lamella, and the node-free valve surface may also help diagnose the new species (Figs 2, 3).

Mazzini et al. (2014) made detailed observations on the morphologies of nine *Ilyocypris* species from Italy and concluded that, although the marginal ripplets are constant within populations of each species, the pattern reported by different authors can be different. Apart from intra-species variability, we think that at least three possible reasons may be responsible for such inconsistencies. Firstly, as fine structures, the marginal ripplets can be concealed by organic material that has not been removed in extant specimens. Secondly, they can be lost or hidden in fossil material due to various reasons including post-mortem abrasion, partial dissolution, chemical precipitation during diagenesis, and/or the tight closure of the carapace. Thirdly, the morphology of the marginal ripplets may sometimes be inaccurately presented in the interpretive drawings. In regard of the above, we propose that the marginal ripplets can still be useful for species identification of *Ilyocypris*, provided that specimens are properly prepared and possible taphonomic artefacts are considered.

Outline analyses indicate that although *I.leptolinea* Wang & Zhai, sp. nov. can be distinguished from *I.innermongolica* by valve shape, its morpho-space exhibits considerable overlap with other *Ilyocypris* species that have living/fossil representatives in Inner Mongolia, namely *I.bradyi*, *I.japonica*, and *I.mongolica* (Figs 6, 7). This indicates that outline analyses are useful but would not be able to distinguish all the *Ilyocypris* species. Therefore, we suggest that a combination of the valve shape (e.g., Mazzini et al. 2014) and the marginal ripplets (e.g., Janz 1994; Mischke et al. 2003), among other morphological features, should be utilised in the species identification of this genus.

The drawback of valve outline is that it is usually a continuous or semi-continuous feature that generates overlapping patterns between species (Figs 6C, 7). Because of the general similarity of the valve shapes of the species in the genus *Ilyocypris* (Meisch 2000; Smith et al. 2019), with the inclusion of more species and more morpho-types into the outline analysis, we would expect that the subtle differences between some of the species may be filled by ‘marginal’ morpho-types of each species, obscuring the inter-species boundary. Nevertheless, outline analysis should still be useful in recognising some of the species, like *I.innermongolica* in our study (Figs 6, 7). We may also be optimistic that, in the future, with the increased feasibility of three-dimensional observation techniques, like 3D scanning and micro-CT, the present-day 2D outline analysis can potentially evolve into 3D-shape analysis, which would capture more shape information for species discrimination.

Most of the valve-shape difference among different *Ilyocypris* species is present in the posterior part of the valves while the shapes of the anterior parts are almost identical (Fig. 6B). This possibly implies that the areas holding the soft parts responsible for walking (the sixth limbs and the uropods), digestion and reproduction exhibit more inter-species variation. Measurement data of the various limb podomeres, chaetotaxy structures, and other soft parts of *Ilyocypris* would be helpful for testing this assumption. However, the hitherto published work of such kind, Zhai et al. (2017: fig. 14), measured only 23 body structures of only two *Ilyocypris* species covered in our study, namely *I.innermongolica* and *I.mongolica*, and this is inadequate for explaining the inter-species shape difference that is accentuated in the posterior part of the valves (Fig. 6B).

### ﻿Palaeoenvironmental implications

The prevalence of lacustrine sediments in the Xiaojinggou Basin indicates the existence of a lake in this area during the late Quaternary. From the lithology of the section XJG2, which is dominated by silt and sand fractions and shows a trend of becoming coarser towards the upper part (Fig. 1D), we suggest that the corresponding environment at the sampling site represented a shallow lake, or littoral to intermediate zone of a large lake, with the water becoming shallower across the studied time span. The ostracod composition of the section lacks the species *Limnocythereinopinata* (Baird, 1843), which dominates the small and large brackish waterbodies in the area nowadays (Zhai et al. 2010, 2013, 2015, 2022). This species was also abundant in the large brackish lakes in eastern Inner Mongolia during the late Quaternary (Shen et al. 2001; Zhai et al. 2011; Yue et al. 2022). This may indicate that the palaeo-lake in Xiaojinggou Basin was freshwater, although *L.inopinata* is known from freshwater environments elsewhere (Meisch 2000). Alternatively, as *L.inopinata* is typical of permanent waterbodies, its absence could be because the lake periodically dried up. Given the close position of the XJG2 section to one of the three largest brackish lakes, Dali, in eastern Inner Mongolia (Fig. 1B), the presence of an ephemeral and/or freshwater lake would be significant for palaeoenvironmental reconstruction of the area. However, before quantitative assemblage data of the ostracods from different intervals of the section are available, it would be difficult to draw more specific inferences on the palaeoenvironmental conditions in this area. Although van Harten (1979) speculated that frequent marginal ripplets on the left valves of *Ilyocypris* (as also occurs in *I.leptolinea* Wang & Zhai, sp. nov.) could denote environmental instability, this assumption has not been tested with modern datasets and is therefore not applied in the present study.

The disappearance of *I.leptolinea* Wang & Zhai, sp. nov. above the depth of 20 cm in the XJG2 section may be significant, perhaps even indicative of its extinction. This species has not been reported from Quaternary sediments elsewhere or from modern habitats, and it may have been endemic to the Xiaojinggou area during the late Quaternary, with a comparatively narrow ecological niche. It would not only add to our knowledge of the animals that went extinct during the Quaternary (Deng et al. 2013; Zhang et al. 2019; Li et al. 2021b), but should also imply certain underlying mechanisms. Whether and how natural or artificial environmental changes have resulted in its extinction, however, will remain unknown before the exact timing of its disappearance and the accompanying changes in fauna, flora, and human activities in the study area can be reconstructed. These issues should be left to future studies.

## ﻿Conclusions

This work is the first investigation of the lacustrine sediments and the ostracod fossils in the Xiaojinggou Basin, and future works will endeavour to reconstruct the palaeoenvironments of this area based on more analyses of the ostracods and other proxies. With previous palaeoenvironmental investigations in eastern Inner Mongolia focused on the three large brackish-water lakes (Xiao et al. 2004, 2008, 2009; Zhai et al. 2011), the discovery of a palaeo-lake in the Xiaojinggou Basin with freshwater conditions would be unique.

Although *Ilyocyprisleptolinea* Wang & Zhai, sp. nov. is ‘ordinary’ among the species of the genus as judged from its carapace size, shape, and not noded, pitted valve surface, the patterns of marginal ripplets and the inner lists on its left valves are unique. The erection of this species based on the observations of various features, especially the marginal ripplets and the outline analyses, could become a case study for describing new species of the genus *Ilyocypris* based on valve material. Judged from the lithology and accompanying ostracods in the section, *I.leptolinea* Wang & Zhai, sp. nov. may have lived in intermediate depth to shallow freshwater, although as an extinct species it could hardly provide independent environmental indications. The new species may add to the knowledge of extinct animals in the late Quaternary and provides basic data for studying environmental changes during this period.

## Supplementary Material

XML Treatment for Ilyocypris leptolinea
